# Neural retina identity is specified by lens-derived BMP signals

**DOI:** 10.1242/dev.123653

**Published:** 2015-05-15

**Authors:** Tanushree Pandit, Vijay K. Jidigam, Cedric Patthey, Lena Gunhaga

**Affiliations:** Umeå Centre for Molecular Medicine, Umeå University, Umeå 901 87, Sweden

**Keywords:** BMP, Chick, Development, Eye, Lens, Neural retina

## Abstract

The eye has served as a classical model to study cell specification and tissue induction for over a century. Nevertheless, the molecular mechanisms that regulate the induction and maintenance of eye-field cells, and the specification of neural retina cells are poorly understood. Moreover, within the developing anterior forebrain, how prospective eye and telencephalic cells are differentially specified is not well defined. In the present study, we have analyzed these issues by manipulating signaling pathways in intact chick embryo and explant assays. Our results provide evidence that at blastula stages, BMP signals inhibit the acquisition of eye-field character, but from neural tube/optic vesicle stages, BMP signals from the lens are crucial for the maintenance of eye-field character, inhibition of dorsal telencephalic cell identity and specification of neural retina cells. Subsequently, our results provide evidence that a *Rax2*-positive eye-field state is not sufficient for the progress to a neural retina identity, but requires BMP signals. In addition, our results argue against any essential role of Wnt or FGF signals during the specification of neural retina cells, but provide evidence that Wnt signals together with BMP activity are sufficient to induce cells of retinal pigment epithelial character. We conclude that BMP activity emanating from the lens ectoderm maintains eye-field identity, inhibits telencephalic character and induces neural retina cells. Our findings link the requirement of the lens ectoderm for neural retina specification with the molecular mechanism by which cells in the forebrain become specified as neural retina by BMP activity.

## Abstract (precis)

**SUMMARY:** BMP signals from the lens are crucial to maintain eye-field character, inhibit dorsal telencephalic cell identity, and specificy neural retina cells in chick embryos.

## INTRODUCTION

During early development of the vertebrate central nervous system (CNS), the anterior neural domain becomes restricted into different regions, giving rise to the telencephalon, eye-field and hypothalamus (reviewed by Garcia-Lopez et al., 2009). The eye-field gives rise to most structures of the eye, such as the neural retina, the retinal pigment epithelium (RPE) and the optic stalk. By contrast, the lens of the eye derives from the lens ectodermal placode (reviewed by Gunhaga, 2011). Whether the specification of eye-field cells and subsequent induction of neural retina cells occur in a single or distinct inductive event, and how this is regulated is not known. Moreover, when and how neural retina cells are specified in relation to other eye and forebrain structures, and whether the prospective lens plays any role in this process, remain to be examined.

When describing development of any cell type, it is important to distinguish between cell fate and cell specification. Cell fate is the identity a cell adopts if left undisturbed in the embryo, whereas cell specification is defined as the step whereby cells have received sufficient signals to acquire a specific cell identity if cultured outside the embryo (Patthey and Gunhaga, 2011). Fate maps of chick embryos at neural plate stages have defined prospective eye-field cells in the anterior neural plate (Fernández-Garre et al., 2002; Sánchez-Arrones et al., 2009). Previous studies have revealed the importance of a set of overlapping transcription factors, such as *Rax*, *Rax2*, *Six3*, *Six6*, *Pax6* and *Lhx2*, in promoting an eye-field identity (Grindley et al., 1995; Lagutin et al., 2003; Swindell et al., 2008; Zuber et al., 2003). The transcription factor genes *Rax* (retinal and anterior neural fold homeobox) and *Rax2* are among the earliest markers of the eye-field, being initially expressed in the anterior neural region of head-fold stage embryos, but later becoming restricted to the neural retina and the ventral hypothalamus (Sánchez-Arrones et al., 2009). Inactivation of *Rax* in mouse or its ortholog *rx3* in zebrafish within the anterior neural plate leads to complete absence of eyes as a result of failure to form the optic vesicles (Loosli et al., 2003; Mathers et al., 1997). However, how *Rax2*-positive eye-field cells progress to a neural retina character has not been determined.

At neural tube closure, the optic vesicle comes in contact with the prospective lens ectoderm. In mammals and birds, the optic vesicle and the lens placode invaginate simultaneously. Following invagination, the optic vesicle transforms into a bilayered optic cup, in which the inner layer gives rise to the neural retina and the outer layer gives rise to the RPE (Fuhrmann, 2010). At optic vesicle stages, *Vsx2* (visual system homeobox 2, previously known as *Chx10*) is upregulated in the prospective neural retina (Fuhrmann, 2010), whereas *Mitf* (microphthalmia associated transcription factor) is induced in the presumptive RPE (Mochii et al., 1998). *Vsx2* mutants exhibit reduced proliferation of neural progenitors within the optic vesicle and, at later stages, bipolar cells are absent from within the retina (Burmeister et al., 1996). The signals that regulate the specification of neural retina cells and when this occurs have not yet been defined. Moreover, whether the specification of neural retina cells requires lens-derived signals and which one(s) remains controversial (Eiraku et al., 2011; Hyer et al., 1998).

Bone morphogenetic protein (BMP) signals have been shown to play important roles during eye formation. Several studies have shown that BMP activity is required for lens induction (Furuta and Hogan, 1998; Pandit et al., 2011; Rajagopal et al., 2009; Sjödal et al., 2007; Wawersik et al., 1999). In addition, in both chick and mouse, BMP activity regulates the initial dorso-ventral patterning of the neural retina (Adler and Belecky-Adams, 2002; Kobayashi et al., 2010; Murali et al., 2005). In mouse, it has also been shown that BMP signaling is essential for retinal growth after embryonic day (E) 10.5 and for early retina neurogenesis (Murali et al., 2005). *In vivo* studies in chick have revealed that BMP activity is required for the development of the RPE (Muller et al., 2007), and that implanted BMP-soaked beads result in downregulation of neural retina markers and induction of RPE-like cells (Hyer et al., 2003; Muller et al., 2007). However, whether BMP signals are involved in the maintenance of eye-field identity and/or specification of neural retina cells has not been determined.

In the present study in chick, we show that eye-field cells become independent of adjacent tissues only at stage 13, coincident with the specification of neural retina cells. Prior to this stage, eye-field and optic vesicle cells cultured alone acquire dorsal telencephalic character. At the blastula stage, low levels of BMP signals prevent the generation of eye-field cells, whereas at neural tube/optic vesicle stages, BMP signals from the lens ectoderm are required and sufficient to maintain eye-field identity, block telencephalic character and specify neural retina cells. In addition, our results argue against any essential role for Wnt or FGF signals during the specification of neural retina cells.

## RESULTS

### Characterization of markers of the optic vesicle and other forebrain domains

**Fig. 1.**
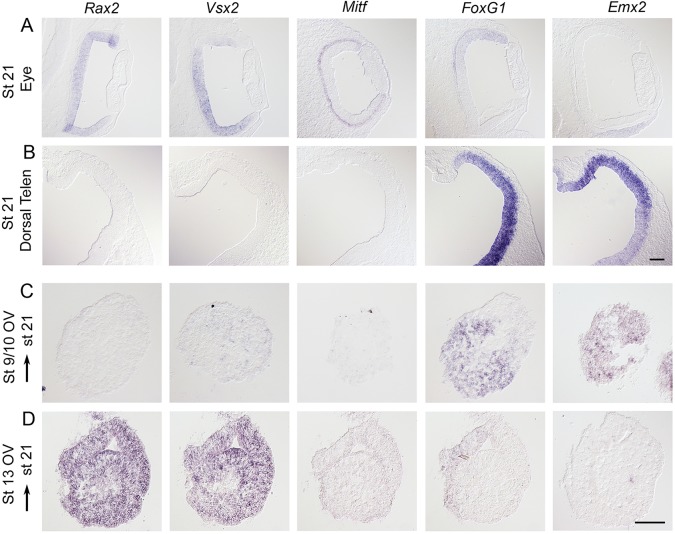
**Expression patterns of optic vesicle and anterior forebrain markers.** (A,B) Expression patterns of various anterior forebrain markers were analyzed at stage 21 by *in situ* hybridization on consecutive sections. (A) At stage 21, *Rax2* and *Vsx2* are expressed in the neural domain, and *Mitf* expression is restricted to the RPE domain of the optic cup. *FoxG1* expression is confined to the dorsal periphery of the optic cup. (B) At stage 21, *FoxG1* and *Emx2* are strongly expressed in the dorsal telencephalon. *Rax2* and *Vsx2* are not expressed in the dorsal telencephalon. (C,D) Optic vesicle (OV) explants cultured to approximately stage 21 and analyzed by *in situ* hybridization on consecutive sections. (C) Stage 9/10 OV explants generated *FoxG1*^+^ (25/25) and *Emx2*^+^ (25/25) dorsal telencephalic cells, but no *Rax2*^+^ (0/25), *Vsx2*^+^ (0/25) or *Mitf*^+^ (0/25) retinal cells. (D) Stage 13 OV explants generated *Rax2*^+^ (15/15) and *Vsx2*^+^ (15/15) neural retina cells, and a few *FoxG1*^+^ cells in a restricted region (0/15), but no *Mitf*^+^ (0/15) RPE cells or *Emx2*^+^ (0/15) cells were detected. Scale bars: 100 µm.

To examine when cells of the eye-field acquire neural retina character, we analyzed the generation of neural retina cells in relation to other eye and forebrain cells. To achieve this, the expression of a panel of markers was monitored in chick (*Gallus gallus*) embryos from stage 9 to stage 21 of development on consecutive sections (Fig. 1; supplementary material Fig. S1).

In stage 9 chick embryos, *Rax2* is expressed in the evaginating optic vesicle and in prospective hypothalamic cells (supplementary material Fig. S1A). At this stage *FoxG1* (previously known as *BF-1*) is expressed in telencephalic cells (supplementary material Fig. S1A). By stage 11, *Rax2* expression is restricted to the prospective optic vesicle (Fig. 1B). From this stage onwards, *FoxG1* expression is detected in the periphery of the optic vesicle in addition to strong expression in the telencephalon (Fig. 1A; supplementary material Fig. S1B,C). At stage 13, *Vsx2* is upregulated, and, in the forebrain, overlapping expression of *Rax2* and *Vsx2* is detected only in the neural domain of the optic vesicle, whereas *Mitf* expression is upregulated in the prospective RPE (supplementary material Fig. S1C). At stage 21, *Rax2* and *Vsx2* continue to be co-expressed only in the neural retina, whereas *Mitf* is expressed in the RPE (Fig. 1D). In addition, *Rax2* is weakly expressed in the tuberal hypothalamus, but *Vsx2* and *Mitf* are not expressed in the forebrain outside the neural retina and RPE, respectively (supplementary material Fig. S1D,E; data not shown). Strong expression of both *FoxG1* and *Emx2* marks the dorsal telencephalon, but no other regions of the forebrain (Fig. 1; supplementary material Fig. S2). Taken together, these results show that neural retina cells can be distinguished from other eye and forebrain cell types by the co-expression of *Rax2* and *Vsx2* from early developmental stages.

### The generation of eye-field cells requires inhibition of BMP signals at blastula stages

**Fig. 2.**
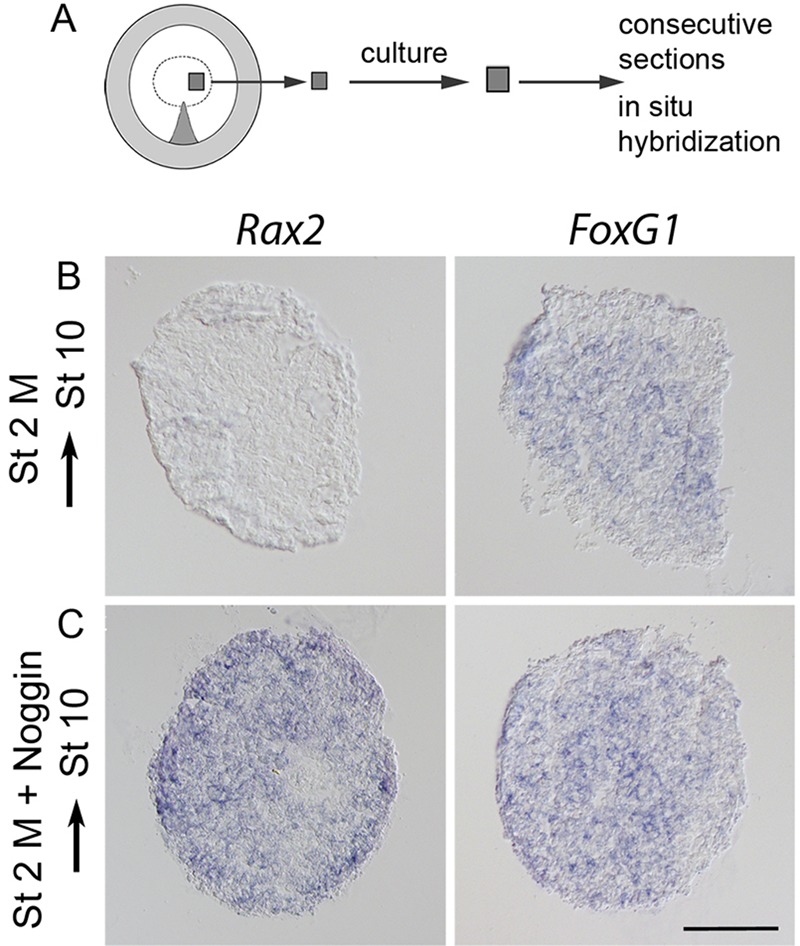
**Eye-field cells are not specified at the blastula stage.** (A) The explant method and location of stage 2 medial (M) explants. (B,C) Stage 2 M explants cultured to approximately stage 10 and analyzed by *in situ* hybridization. (B) Stage 2 M explants generated *FoxG1*^+^ (8/15) cells, but no *Rax2*^+^ (0/15) eye-field cells. (C) In stage 2 M explants cultured together with Noggin, *Rax2*^+^ (14/17) eye-field and *FoxG1*^+^ (16/17) cells were generated. Scale bar: 100 µm.

A recent study in zebrafish has suggested that at blastula to gastrula stages, BMP signals in the anterior neural ectoderm promote telencephalic identity at the expense of eye-field character (Bielen and Houart, 2012). To examine whether this molecular mechanism also acts in amniotes, we isolated late blastula stage (stage 2) medial (M) explants (Fig. 2A; Patthey et al., 2009) and cultured them alone or in the presence of the BMP inhibitor Noggin for 33 h, by which time intact embryos would correspond to approximately stage 10. Thereafter, the explants were processed and analyzed for marker expression on consecutive sections (Fig. 2A; see Materials and Methods for details).

Stage 2 M explants are specified as dorsal telencephalic cells (Patthey et al., 2009). Accordingly, stage 2 M explants cultured alone generated *FoxG1*^+^ cells, but no *Rax2*^+^ cells (Fig. 2B). By contrast, stage 2 M explants cultured together with Noggin generated *Rax2*^+^ and *FoxG1*^+^ cells (Fig. 2C). Taken together, these results indicate that: (1) eye-field cells are not specified at the late blastula stage; and (2) BMP activity represses eye-field character at blastula stages and needs to be inhibited in prospective forebrain cells for the generation of *Rax2*^+^ eye-field cells.

### Isolated eye-field cells switch to dorsal telencephalic identity during culture

To determine whether the specification of eye-field cells and neural retina cells occur in a single or distinct inductive event, we tested whether eye-field cells differentiate into *Rax2*^+^ and *Vsx2*^+^ neural retina cells in culture. Guided by fate maps (Garcia-Lopez et al., 2009) and optic vesicle morphology (supplementary material Fig. S3), we isolated optic vesicle (OV) explants from stage 9, 10, 11 and 13 embryos. In accordance with *in vivo* expression patterns (supplementary material Fig. S1A-C; Bell et al., 2001), at the onset (0 h) of culture the majority of stage 9, 10, 11 and 13 explants consisted of *Rax2*^+^ cells and a few *FoxG1*^+^ cells at one edge of the explants (supplementary material Fig. S4A-D) but no *Emx2*^+^ cells (data not shown). Thus, OV explants at stage 9 to 13 consist mainly of eye-field cells.

Unexpectedly, *Rax2* expression was not maintained in stage 9, 10 or 11 OV explants, and no *Vsx2*^+^ cells or *Mitf*^+^ cells were induced when cultured for 46-54 h (∼stage 21) (Fig. 1C; data not shown). Instead, stage 9-11 OV explants generated *FoxG1*^+^ and *Emx2*^+^ cells (Fig. 1C; data not shown), which is characteristic of a dorsal telencephalic identity (Gunhaga et al., 2003; Martynoga et al., 2005; von Frowein et al., 2006) (Fig. 1B). The absence of lens fiber cells in the cultured OV explants was verified by the lack of δ-crystallin^+^ cells (Pandit et al., 2011; Shinohara and Piatigorsky, 1976; data not shown). Thus, at stage 9-11, isolated and cultured optic vesicle cells acquire a dorsal telencephalic character.

By contrast, stage 13 OV explants generated *Rax2*^+^ and *Vsx2*^+^ neural retina cells and a few *FoxG1*^+^ cells, but no *Mitf*^+^ RPE cells or *Emx2*^+^ cells after 38-40 h culture (Fig. 1D), implying that neural retina cells are specified at stage 13. Taken together, these data provide evidence that, until stage 13, maintenance of Rax2-positive eye-field identity and induction of neural retina cells requires signals from adjacent tissues.

### The generation of retinal cells is dependent on the lens ectoderm and on BMP signals

**Fig. 3.**
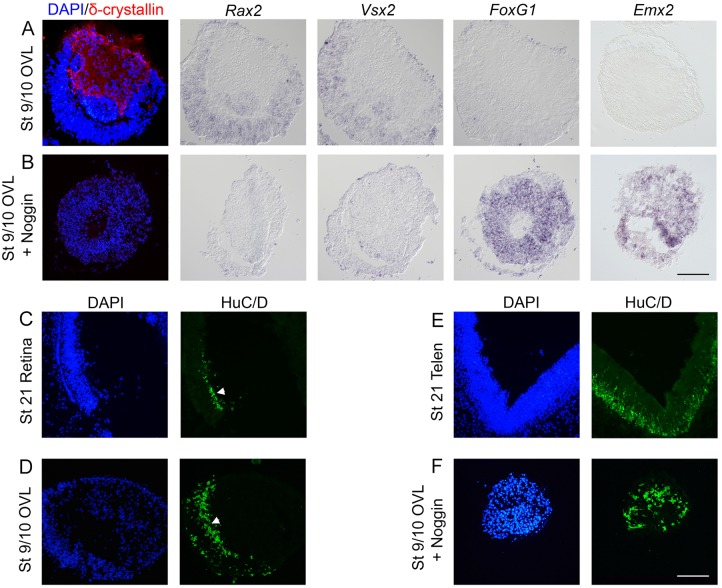
**Neural retina cell identity is dependent on BMP signals and the lens ectoderm.** (A,B,D,F) Stage 9 and 10 optic vesicle/prospective lens (OVL) explants cultured to approximately stage 21 and analyzed by immunohistochemistry and *in situ* hybridization. (A) Stage 9/10 OVL explants generated *Rax2*^+^ (25/25) and *Vsx2*^+^ (25/25) neural retina cells, and δ-crystallin^+^ (25/25) lens cells in non-overlapping regions of the explants. No *FoxG1*^+^ (0/25) or *Emx2*^+^ (0/25) dorsal telencephalic cells were detected. (B) In stage 9/10 OVL explants cultured together with Noggin, the generation of *Rax2*^+^ (0/25) and *Vsx2*^+^ (0/25) retinal cells was inhibited in the prospective retinal compartment, and cells acquired a *FoxG1*^+^ (25/25) and *Emx2*^+^ (25/25) dorsal telencephalic cell identity*.* In the prospective lens domain of the OVL explants, the generation of δ-crystallin^+^ (0/25) lens cells was blocked. (C) HuC/D expression in the neural retina at stage 21 (white arrowhead). (D) Stage 9/10 OVL explants generate a regularly aligned pattern of HuC/D^+^ cells within the retinal domain of the explants (white arrowhead) (15/15). (E) HuC/D expression in the telencephalon at stage 21. (F) In stage 9/10 OVL explants cultured together with Noggin, HuC/D^+^ cells are scattered in a telencephalon-like pattern (15/15). Scale bars: 100 µm.

At stages 10-13, the prospective lens ectoderm lies in close apposition to the developing optic vesicle (supplementary material Fig. S1B,C). To determine whether the lens ectoderm is sufficient to specify neural retina cells, we cultured stage 9/10 optic vesicle cells together with prospective lens ectoderm (OVL explants) for 52-54 h (∼stage 21). In cultured OVL explants, *Rax2*^+^ and *Vsx2*^+^ cells, which are characteristic of the neural retina, and δ-crystallin^+^ cells, which are characteristic of lens fiber cells, were generated in distinct non-overlapping regions of the explants (Fig. 3A). No *FoxG1*^+^ and *Emx2*^+^ dorsal telencephalic cells (Fig. 3A) or *Mitf*^+^ RPE cells (data not shown) were generated. Thus, in the presence of prospective lens ectoderm, optic vesicle cells maintain the expression of *Rax2* and acquire neural retina identity.

We and others have previously shown that around stage 11, *Bmp4* is expressed in the prospective lens ectoderm (Pandit et al., 2011; Trousse et al., 2001) and that phosphorylated Smad1 (pSmad1) is enriched in the optic vesicle (Belecky-Adams et al., 2002). These data imply that BMP signals emanating from the lens are important for the activation of the BMP pathway in the optic vesicle and subsequent development of the neural retina. To examine this hypothesis, we cultured stage 9/10 OVL explants in the presence of Noggin. After BMP inhibition, the generation of *Rax2*^+^ and *Vsx2*^+^ neural retina cells was suppressed and, instead, *FoxG1*^+^ and *Emx2*^+^ dorsal telencephalic cells were induced (Fig. 3B). Moreover, the spatial organization of the generated HuC/D^+^ post-mitotic neurons changed from a regularly aligned pattern that is similar to that shown by retina neurons *in vivo* (Fig. 3C,D), to a scattered telencephalon-like pattern throughout the CNS-derived part of the OVL explants (Fig. 3E,F). In addition, and in agreement with our previous publications (Pandit et al., 2011; Sjödal et al., 2007), the generation of δ-crystallin^+^ lens cells was blocked by BMP inhibition (Fig. 3A,B). These results suggest that BMP signals emanating from the prospective lens ectoderm are required for proper specification of neural retina cells, and in the absence of BMP activity, eye-field cells acquire dorsal telencephalic identity.

### Lens-derived BMP signals are required for neural retina development in intact chick embryos

To examine the requirement for BMP signaling during the specification of neural retina cells *in vivo*, chick embryos were electroporated *in ovo* in the optic vesicle area (stage 9/10) to transfer a green fluorescent protein (GFP) vector alone or together with a Noggin-expressing vector (Timmer et al., 2002). The electroporated embryos were cultured to approximately stage 15-16, and embryos with GFP staining within the retina region were selected for further analyses. The optic vesicle region of Noggin-electroporated embryos was compared with the corresponding domain of the non-electroporated side as well as control GFP-electroporated embryos.

**Fig. 4.**
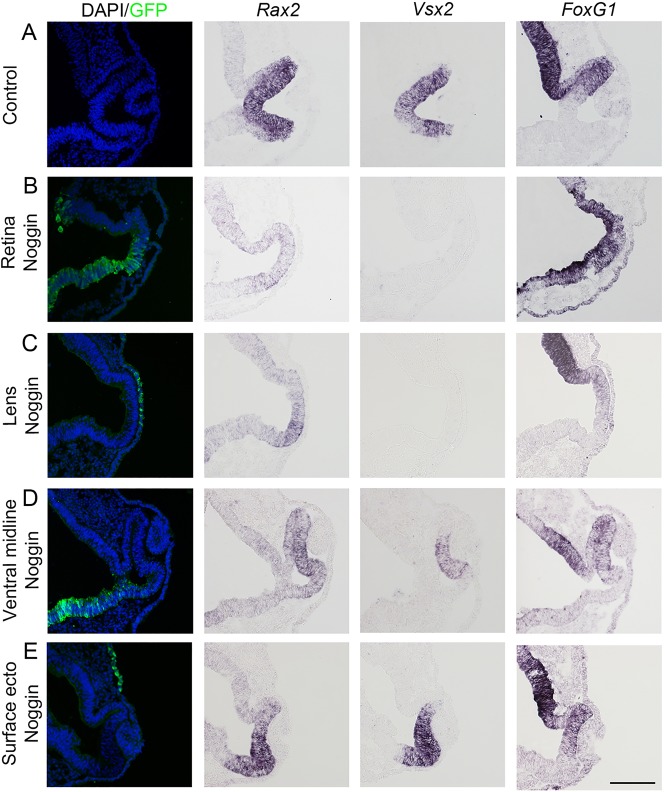
**Failure of retina development *in vivo* after BMP inhibition.** *In ovo* electroporation of stage 9/10 embryos in the optic vesicle area or surrounding tissues using a GFP and a Noggin vector was performed. Then embryos were cultured to stage 15/16 and analyzed by immunohistochemistry and *in situ* hybridization on consecutive sections. (A) Control non-electroporated retina with expression of *Rax2* and *Vsx2* in the neural retina, and weak expression of *FoxG1* in the dorsal part of the neural retina (*n*=8). (B,C) Noggin-electroporation in the optic vesicle resulted in a non-invaginated circular-shaped retina (8/8). The expression of *Rax2* was downregulated and *Vsx2* expression was completely lost or inhibited, whereas the expression of *FoxG1* was expanded (8/8). (C) Noggin-electroporation in the prospective lens ectoderm resulted in a non-invaginated circular-shaped retina (8/8). *Vsx2* expression was clearly inhibited and *Rax2* expression was downregulated, whereas the expression of *FoxG1* appeared unchanged (8/8). (D,E) No change in retina morphology or expression of *Rax2*, *Vsx2* and *FoxG1* was observed after Noggin was electroporated in the ventral midline of the forebrain (*n*=4) or in the head ectoderm outside of the prospective lens ectoderm (*n*=4). Scale bar: 100 µm.

All control GFP-electroporated embryos and control sides of the Noggin-electroporated embryos exhibited normal morphology of the lens and retina, and a normal expression pattern of *Rax2*, *Vsx2* and *FoxG1* (Fig. 4A; supplementary material Fig. S5). By contrast, in embryos with Noggin-electroporated optic vesicles, the prospective retina failed to invaginate and did not form a bilayered structure (Fig. 4B). Moreover, the expression of *Vsx2* was lost or severely inhibited, and *Rax2* expression was downregulated in the malformed circular-shaped retina, whereas the expression of *FoxG1* was expanded (Fig. 4B). In addition, the lens also failed to invaginate and did not form a proper vesicle (Fig. 4B). Thus, BMP activity is required for the early development of the neural retina *in vivo*.

To evaluate potential sources of BMP signals required for the specification of neural retina cells *in vivo*, we electroporated the Noggin construct in stage 9/10 chick embryos in different locations: (1) in the prospective lens ectoderm; (2) in the head ectoderm outside of the prospective lens ectodermal region; and (3) in the ventral midline of the forebrain. When the Noggin construct was electroporated in the prospective lens ectoderm, the optic vesicle failed to invaginate and did not form a bilayered structure (Fig. 4C). Moreover, the induction of *Vsx2* expression was lost or severely inhibited, and *Rax2* expression was downregulated in the malformed eye region, although no obvious change in *FoxG1* expression was observed (Fig. 4C). By contrast, when Noggin was electroporated in the ventral midline of the forebrain or in the head ectoderm, no change in eye morphology or expression of *Rax2* and *Vsx2* in the neural retina was observed (Fig. 4D,E; supplementary material Fig. S5C,D). These data suggest that lens-derived BMP signals are required for the maintenance of eye-field identity and the induction of neural retina cells.

### Direct requirement of BMP activity for the specification of neural retina cells

To test whether the specification of neural retina cells requires BMP signaling directly in the optic vesicle independently of the lens ectoderm, we used stage 13 OV explants that generate *Rax2*^+^ and *Vsx2*^+^ neural retina cells during culture in the absence of lens cells (Fig. 1D). Stage 13 OV explants cultured in the presence of Noggin all exhibited the same pattern: in half of the explant, the generation of *Rax2*^+^ and *Vsx2*^+^ neural retina cells was completely abolished and *FoxG1* expression was upregulated (supplementary material Fig. S6); in the other half of the explant, the expression of *Rax2* was strongly reduced and was combined with no or weak *Vsx2* expression and no detectable *FoxG1* expression (supplementary material Fig. S6). *Emx2* expression was not detected in the Noggin-exposed stage 13 OV explants (supplementary material Fig. S6). This expression profile suggests that cells in half of the explants acquired the identity of *FoxG1*^+^ forebrain cells, although distinct from dorsal telencephalic identity, and cells in the other half acquired an expression profile reminiscent of the tuberal hypothalamus (supplementary material Fig. S1E). Regardless of the acquired cell identity, these results show that ongoing BMP activity is required directly for the specification of neural retina cells.

### BMP activity induces *Fgf8* expression in neural retina cells, but FGF activity is not required or sufficient to specify neural retina identity

**Fig. 5.**
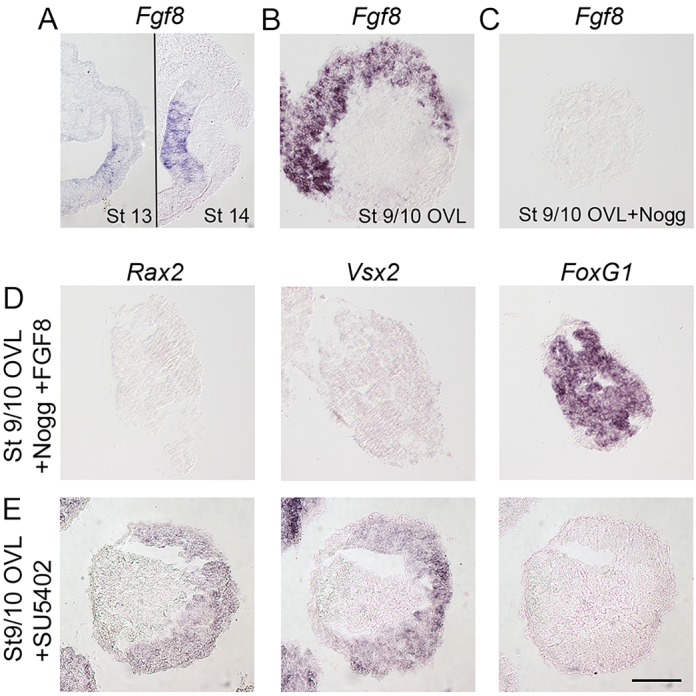
**FGF signals are neither required nor sufficient to induce a neural retina character.** (A) *Fgf8* expression is upregulated within the neural retina at stage 13-14 (stages demarcated by black line). (B-E) Stage 9/10 OVL explants were cultured to approximately stage 21 and analyzed by *in situ* hybridization on consecutive sections. (B) Stage 9/10, OVL explants generate *Fgf8*^+^ (15/15) cells within the retinal domain. (C) In stage 9/10 OVL explants cultured together with Noggin, *Fgf8* (0/15) expression was inhibited. (D) In the presence of Noggin and FGF8, stage 9/10 OVL explants still generated *FoxG1*^+^ (12/12) telencephalic cells, but did not generate *Rax2^+^* (0/12) or *Vsx2^+^* (0/12) neural retina cells. (E) In stage 9/10 OVL explants cultured together with SU5402, the generation of *Rax2^+^* (12/12) and *Vsx2^+^* (12/12) neural retina cells was not inhibited. No *FoxG1* (0/12) expression was observed. Scale bar: 100 µm.

FGF signals have been shown to play a role in the development and patterning of the neural retina (Hyer et al., 1998; Martinez-Morales et al., 2005; Pittack et al., 1997), and in chick embryos *Fgf8* is upregulated in the medial part of the neural retina at stage 13-14 (Fig. 5A). Therefore, we addressed whether FGF signals act together with BMP activity during the specification of neural retina cells. During culture, stage 9/10 OVL explants generated *Fgf8*^+^ cells in the *Vsx2^+^* and *Rax2^+^* domain of the explants (Fig. 5B). By contrast, in the presence of Noggin, *Fgf8* expression was inhibited in prospective retinal cells (Fig. 5C). Thus, inhibition of BMP activity in optic vesicle and lens co-cultures suppresses the upregulation of *Fgf8* in neural retina cells, consistent with a loss of neural retina identity.

To test whether the loss of *Rax2* and *Vsx2* expression observed after BMP inhibition is a secondary effect due to downregulated FGF activity, we cultured stage 9/10 OVL explants together with Noggin and FGF8 (250 ng/ml). Addition of FGF8 could not rescue the generation of *Rax2*^+^ and *Vsx2*^+^ neural retina cells in Noggin-treated explants, and cells still acquired a *FoxG1*^+^ telencephalic identity (Fig. 5D). In agreement with this, exposure to SU5402 (5 µM), an inhibitor of FGF signaling (Mohammadi et al., 1997), did not block the generation of *Rax2*^+^ and *Vsx2*^+^ neural retina cells or promote *FoxG1* expression in stage 9/10 OVL explants (Fig. 5E). Thus, FGF activity is neither required nor sufficient to induce *Rax2*^+^ or *Vsx2*^+^ neural retina cells.

### BMP signals can replace the function of the lens ectoderm to suppress dorsal telencephalic identity and to promote neural retina character

**Fig. 6.**
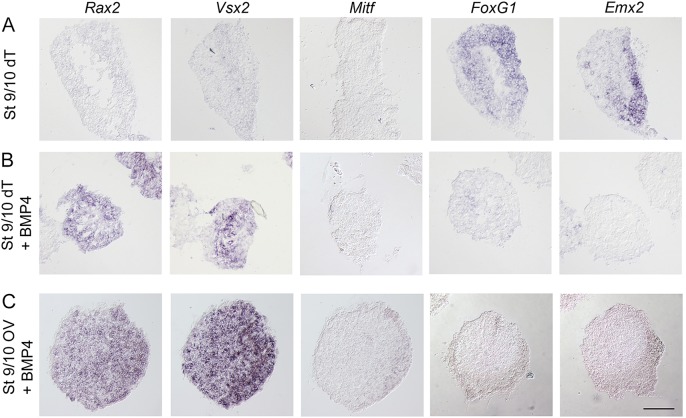
**BMP signals inhibit dorsal telencephalic identity and induce neural retina character.** Stage 9/10 OV and dT explants cultured to approximately stage 21 and analyzed by *in situ* hybridization. (A) In stage 9/10 OV explants, BMP4 (3.5 ng/ml) suppressed the generation of *FoxG1*^+^ (0/20) and *Emx2*^+^ (0/20) dorsal telencephalic cells, and induced *Rax2*^+^ (20/20) and *Vsx2*^+^ (20/20) neural retina cells, but no *Mitf*^+^ (0/20) RPE cells. (B) Stage 9/10 dT explants generated *FoxG1*^+^ (15/15) and *Emx2*^+^ (15/15) cells. No *Rax2*^+^ (0/15), *Vsx2*^+^ (0/15) or *Mitf*^+^ (0/15) retinal cells were detected. (C) In stage 10 dT explants, BMP4 (7-10 ng/ml) suppressed the generation of *FoxG1*^+^ (0/12) and *Emx2*^+^ (0/12) telencephalic cells. *Rax2*^+^ (12/12) and *Vsx2*^+^ (12/12) neural retina cells were induced, but no *Mitf*^+^ (0/12) RPE cells were detected. Scale bar: 100 µm.

Next, we analyzed whether BMP activity is sufficient to suppress dorsal telencephalic cell identity and induce retinal character by culturing stage 9/10 prospective dorsal telencephalic (dT) explants (Gunhaga et al., 2003) alone or in the presence of BMP4. Stage 9/10 dT explants cultured alone generated *FoxG1*^+^ and *Emx2*^+^ dorsal telencephalic cells, but no *Rax2*^+^ or *Vsx2*^+^ neural retina cells or *Mitf*^+^ RPE cells (Fig. 6A). In the presence of BMP4, the generation of *FoxG1*^+^ and *Emx2*^+^ telencephalic cells was completely inhibited in stage 9/10 dT explants, and instead *Rax2*^+^ and *Vsx2*^+^ neural retina cells were induced, but no *Mitf*^+^ RPE cells were detected (Fig. 6B). Thus, BMP activity is sufficient to suppress dorsal telencephalic cell fate and induce *Rax2*^+^ and *Vsx2*^+^ neural retina cells.

To further assess whether BMP signals are sufficient to induce neural retina character, stage 9/10 OV explants, which, when cultured in neutral conditions, generate dorsal telencephalic cells (Fig. 2C), were exposed to low levels of BMP4 during culture. Exposure to BMP4 blocked the generation of *FoxG1*^+^ and *Emx2*^+^ dorsal telencephalic cells, and induced *Rax2*^+^ and *Vsx2*^+^ neural retina cells, but no *Mitf*^+^ RPE cells (Fig. 6C). We next examined whether higher levels of BMP signaling could induce *Mitf*^+^ RPE cells. However, even in the presence of a tenfold higher concentration of BMP4, *Rax2*^+^ and *Vsx2*^+^ neural retina cells, but no *Mitf*^+^ RPE cells, were induced (supplementary material Fig. S7). These data show that in isolated eye-field cells, BMP ligands can substitute for the missing lens ectoderm, and provide evidence that low levels of BMP activity are sufficient to induce neural retina identity.

### Wnt activity is not required or sufficient to specify neural retina cells, but a combination of Wnt and BMP signals induces RPE cells

Wnt signals have been suggested to be important for the generation of RPE cells (Fujimura et al., 2009; Steinfeld et al., 2013), but whether Wnt activity plays any role in the specification of neural retina cells has not been determined. We therefore first tested whether Wnt activity is required for the specification of neural retina cells, by culturing stage 10 OVL explants and stage 13 OV explants together with a soluble Frizzled receptor (Frizzled-conditioned medium) to inhibit Wnt activity (Gunhaga et al., 2003; Hsieh et al., 1999). Stage 10 OVL explants cultured alone or in the presence of Frizzled generated *Rax2*^+^ and *Vsx2*^+^ neural retina cells, whereas no *FoxG1*^+^ cells were detected (supplementary material Fig. S8A,B). Consistently, stage 13 OV explants cultured alone or in the presence of Frizzled also generated *Rax2*^+^ and *Vsx2*^+^ neural retina cells, and a few *FoxG1*^+^ cells in a restricted region (supplementary material Fig. S8C,D). Thus, Wnt signals are not required for the specification of neural retina cells.

**Fig. 7.**
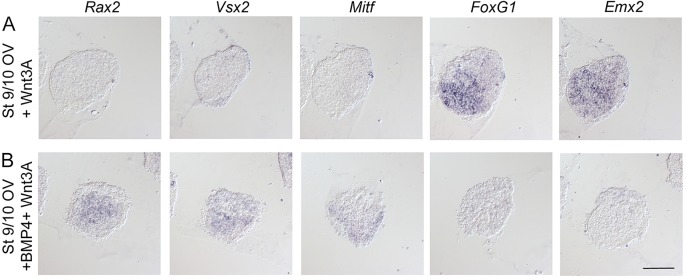
**Combined Wnt and BMP signals induce cells of RPE identity.** Stage 9/10 OV explants cultured to approximately stage 21 and analyzed by *in situ* hybridization. (A) Stage 9/10 OV explants cultured in the presence of Wnt3A still generated *FoxG1*^+^ (12/12) and *Emx2*^+^ (12/12) dorsal telencephalic cells, but no *Rax2*^+^ (0/12), *Vsx2*^+^ (0/12) neural retina cells or *Mitf*^+^ (0/12) RPE cells. (B) Stage 9/10 OV explants cultured together with Wnt3A and BMP4 (3.5 ng/ml) suppressed the generation of *FoxG1*^+^ (0/12) and *Emx2*^+^ (0/12) telencephalic cells, and induced *Mitf*^+^ (0/12) RPE cells, as well as *Rax2*^+^ (12/12) and *Vsx2*^+^ (12/12) neural retina cells. Scale bar: 100 µm.

Next, we tested whether Wnt activity alone or in combination with BMP signals is sufficient to induce neural retina cells or RPE cells, by culturing stage 9/10 OV explants together with Wnt3A-conditioned medium alone or together with low levels of BMP4. Stage 9/10 OV explants cultured together with Wnt3A still generated *FoxG1*^+^ and *Emx2*^+^ dorsal telencephalic cells, but no *Rax2*^+^ or *Vsx2*^+^ neural retina cells or *Mitf*^+^ RPE cells (Fig. 7A). By contrast, the combination of Wnt and BMP activity inhibited the generation of *FoxG1*^+^ and *Emx2*^+^ dorsal telencephalic cells, and induced *Mitf*^+^ RPE cells, as well as *Rax2*^+^ and *Vsx2*^+^ neural retina cells (Fig. 7B). Thus, Wnt activity is not required or sufficient to specify neural retina cells, but a combination of Wnt and BMP signals can induce RPE cells.

## DISCUSSION

In the present study, we have analyzed when and by what mechanisms anterior neural cells become specified as eye-field cells and neural retina cells. In summary, our results provide evidence that, around blastula stages, BMP signals repress the eye-field lineage, and that by early neural tube stages, lens-derived BMP activity maintains eye-field identity, inhibits telencephalic character and induces neural retina cells in the forebrain anlage.

Fate maps in chick have shown that prospective telencephalic cells and prospective retinal cells are situated in close proximity in the anterior neural plate (Cobos et al., 2001; Couly and Le Douarin, 1987; Sánchez-Arrones et al., 2009). Previous results in chick suggest that telencephalic cells of dorsal character are already specified by the late blastula stage (Patthey et al., 2009), and that ventral and definitive dorsal telencephalic identity are specified at the gastrula and early neural tube stages, respectively (Gunhaga et al., 2000, 2003). Using both retina and telencephalic markers, our results now provide evidence that *Rax2*-positive eye-field cells become independent of signals from adjacent tissues at stage 13, which coincides with the specification of neural retina cells. Our data show that, prior to this stage, eye-field cells acquire dorsal telencephalic character when cultured alone. Our study and others (Sánchez-Arrones et al., 2009) have shown that *Rax2* is expressed in the prospective forebrain at early neural tube stages (stage 9/10 in chick) and appears to be a crucial factor in the cell choice between eye and telencephalic identity. Cell tracing experiments performed in the zebrafish *rx3-*null mutants have provided evidence that *rx3*-deficient retinal precursors acquire a telencephalic identity, and embryos exhibit an enlarged telencephalon and lack of eyes (Stigloher et al., 2006). Moreover, a recent study in *Xenopus* has also shown that *Rax* mutant embryos are eyeless and that tissue normally fated to form the retina acquires characteristics of the diencephalon and telencephalon instead (Fish et al., 2014). However, although a *Rax2*-expressing region of the forebrain is devoted to form the neural retina, our results provide evidence that a *Rax2*^+^ eye-field state is not sufficient for progression to a neural retina identity. Thus, prior to stage 13, additional signals from neighboring tissues are required for maintaining *Rax2*^+^ eye-field identity and inducing neural retina character.

During early neural tube stages, the evagination of the eye-field brings the optic vesicle in close contact with the prospective lens ectoderm. Our results show that optic vesicle cells cultured together with the prospective lens ectoderm maintain the *Rax2*^+^ state and upregulate neural retina identity. Moreover, our data suggest that after stage 13, neural retina cells develop independently of signals from the lens ectoderm. In agreement with this, surgical ablation of the lens placode prior to stage 13 resulted in failure of optic cup formation (Hyer et al., 1998), whereas ablation of the lens placode after stage 13 resulted in intact optic vesicles that initiated neural retina differentiation (Hyer et al., 2003). Moreover, in *Pax6* lens-specific mutants, in which lens induction occurs but further lens development is arrested, the differentiation of the neural retina proceeds as normal (Ashery-Padan et al., 2000). These results support our finding that signals from the prospective lens ectoderm are required for maintaining eye-field cells and for inducing neural retina cells.

**Fig. 8.**
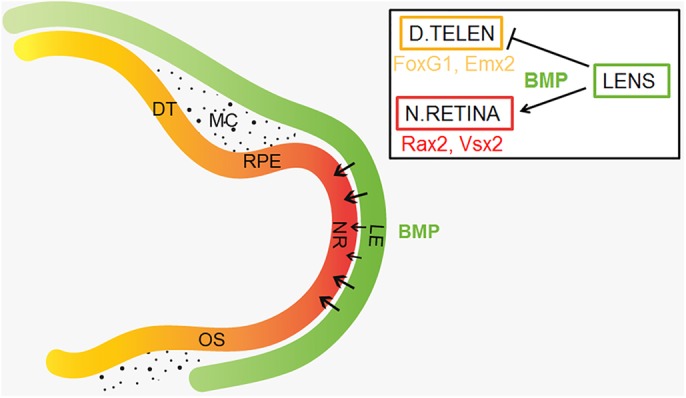
**Neural retina specification in response to lens-derived BMP signals.** At early neural tube to optic vesicle stages, the prospective lens ectoderm is located close to the optic vesicle. Neural retina cells are specified in response to BMP signals, which emanate from the prospective lens ectoderm. Prospective RPE cells are in contact with both the head ectoderm and neural crest-derived mesenchymal cells, whereas the prospective dorsal telencephalic cells are separated from the BMP-rich head ectoderm by neural crest-derived mesenchymal cells. At this stage, BMP activity suppresses dorsal telencephalic identity. DT, dorsal telencephalon; LE, lens ectoderm; MC, mesenchymal cells; NR, neural retina; OS, optic stalk; RPE, retinal pigmented epithelium.

Our *in vitro* gain and loss of BMP function results provide evidence that BMP signals are both required and sufficient to maintain *Rax2*^+^ identity, block dorsal telencephalic character and induce neural retina differentiation. Consistent with this, in retina-specific conditional BMP receptor knockout mice, *Vsx2* failed to be expressed in the mutant optic vesicle and retinal neurogenesis was suppressed (Murali et al., 2005). In addition, our *in ovo* BMP inhibition experiments in various forebrain tissues, suggest that BMP signals from the lens are required for induction of neural retina identity in the optic vesicle. Our finding links the requirement of the lens ectoderm for neural retina specification with the molecular mechanism by which cells in the forebrain become specified as neural retina by BMP activity. Although the optic vesicle and the lens ectoderm are in close contact with each other during early neural tube stages, the adjacent dorsal telencephalon is kept at a distance from the BMP-rich surface ectoderm by intervening neural crest-derived mesenchyme (Fig. 8). The cephalic neural crest cells express the endogenous BMP inhibitors *Noggin* and *Gremlin* (Creuzet, 2009), implying that the cephalic neural crest cells might act as a physical and molecular barrier to protect dorsal telencephalic identity from head-ectoderm-derived BMP signals (Fig. 8). In agreement with this, a recent study in chick has provided evidence that a reduction in *Noggin* and *Gremlin* expression in the cephalic neural crest cells results in abolished *FoxG1* expression in the telencephalon and subsequent microcephaly and partial holoprosencephaly (Aguiar et al., 2014). Our results, showing that BMP signals inhibit telencephalic identity while promoting neural retina character, highlight a new role for BMP signals during the development of the retina that is distinct from previously described roles in dorso-ventral patterning of the retina (Adler and Belecky-Adams, 2002; Kobayashi et al., 2010; Murali et al., 2005) and fate choice between neural versus RPE cells (Hyer et al., 2003; Muller et al., 2007).

It is noteworthy that the lens ectoderm and optic vesicle are so tightly attached that enzymatic treatments are required to dissect the two tissues apart (see Material and Methods). The tight connection can in part be explained by the existence of F-actin-rich filopodia protruding from the lens ectoderm to the prospective neural retina epithelium, which have been shown to be important for proper development of the eye (Chauhan et al., 2009). Specialized filopodia, termed cytonemes, have recently been suggested to function as conduits for morphogen dispersion between morphogen-producing cells and their target tissue (reviewed by Kornberg and Roy, 2014). Thus, existing filopodia might enable the transfer of BMP molecules between the prospective lens and the optic vesicle.

Our results show that, regardless of concentration, BMP activity is not sufficient to induce *Mitf*^+^ RPE cells in prospective optic vesicle explants. In addition, optic vesicle and lens co-cultures did not generate any *Mitf*^+^ RPE cells, indicating that additional signal(s) from other tissues than the lens ectoderm are required for the specification of RPE cells. A previous finding that BMP4-soaked beads implanted under the optic vesicle at stage 10-12 resulted in pigmentation in the entire optic vesicle (Hyer et al., 2003), does not exclude the possibility that BMP activity in combination with other signals from surrounding tissues induces ectopic pigmented cells. Consistent with this, other studies have suggested that the extra-ocular mesenchyme, which surrounds the prospective RPE domain (Fig. 8), is a source of RPE-inducing signals (Fuhrmann et al., 2000; Kagiyama et al., 2005). Our results suggest that a combination of BMP and Wnt activity is sufficient to induce *Mitf*^+^ RPE cells. This is in agreement with previous studies showing that both BMP and Wnt signals are required for the induction of RPE cells, and that neither BMP nor Wnt activity alone is sufficient to induce RPE identity (Steinfeld et al., 2013; Veien et al., 2008).

Our results provide evidence that the specification of neural retina cells is independent of FGF activity. Consistently, the early generation of retina cells appears to be unaffected in *Fgf1*, *Fgf2* and *Fgf1/Fgf2* double-knockout mice (Miller et al., 2000). Furthermore, our results show that inhibition of BMP activity in optic vesicle and lens co-cultures suppresses expression of *Fgf8* in prospective retinal cells, and that simultaneous ectopic addition of FGF8 cannot rescue retinal identity. In agreement with this, in *Bmp4* mouse mutants and in mice that lack *Bmpr1a* and *Bmpr1b* in the retina, *Fgf15* expression is drastically reduced or absent in the optic vesicle (Murali et al., 2005). Moreover, the *Fgf15* expression is restored by the application of BMP4-soaked beads in explanted *Bmp4*^−/−^ optic vesicles (Murali et al., 2005). These results support our conclusion that BMP signals, but not FGF activity, are required and sufficient to induce cells of neural retina identity.

Our results show that the role BMP signals play for the generation of eye-field cells changes between the blastula and neural tube stages. We find that endogenous BMP activity in neural tissue isolated at blastula stages inhibits *Rax2* expression, without being required for *FoxG1* expression. This is consistent with a recent study in zebrafish showing that, at late blastula to gastrula stages, BMP activity in the anterior neural ectoderm suppresses eye-field identity through the inhibition of Rx3, a homolog of Rax2 (Bielen and Houart, 2012). In addition, our previous studies at the blastula stage show that ectopic BMP signals induce epidermal character in prospective neural cells (Patthey et al., 2009). Taken together, this suggests that, at early stages, endogenous low levels of BMP signaling in the anterior neural plate are important for protecting prospective telencephalic cells from acquiring an eye-field identity, whereas, at neurula stages, BMP signaling from the lens ectoderm promotes neural retina identity at the expense of a telencephalic character.

Studies from 3D stem cell differentiation assays have confusingly suggested that neural stem cells spontaneously generate cells of retinal identity (Eiraku et al., 2011) or cerebral cortical (dorsal telencephalic) character (Lancaster et al., 2013). In light of our model, it is possible that the presence or absence of low levels of endogenous BMP signals in these assays directs the generation of retinal versus cerebral cortical cells. Consistently, in human embryonic stem cell cultures eye-field and neural retina markers are rarely upregulated during conditions of constitutive BMP inhibition, whereas telencephalic markers are significantly enhanced (Lupo et al., 2013). Therefore, any protocols developed for the differentiation of stem cells into retina cells should take into account that in the embryonic forebrain anlage, neural retina cells are specified by BMP signals around optic vesicle stages.

## MATERIALS AND METHODS

### Embryos

Fertilized White Leghorn chicken eggs were obtained from Strömbäcks Ägg, Umeå, Sweden. Chick embryos were staged according to the protocol of Hamburger and Hamilton (1951).

### Explant assay

Medial (M) explants were isolated from stage 2 chick embryos (Patthey et al., 2009). Prospective optic vesicle (OV) and optic vesicle/lens (OVL) explants were isolated from stage 9, 10, 11 and 13 chick embryos. Dorsal telencephalic (dT) explants were dissected from stage 10 chick embryos (Gunhaga et al., 2003). Explants were cultured in serum-free conditions with or without addition of factors. For details regarding the explant cultures, culture medium and factors, see the methods in the supplementary material.

### *In ovo* electroporation

Stage 9/10 embryos were electroporated in tissues of interest. Noggin-electroporated tissues and the control non-electroporated side were compared with regions electroporated with GFP only. For vectors used and electroporation settings, see the methods in the supplementary material.

### *In situ* hybridization and immunohistochemistry

*In situ* RNA hybridization and immunohistochemistry were performed essentially as described previously (Wilkinson and Nieto, 1993; Wittmann et al., 2014). Probes and antibodies are described in the methods in the supplementary material.

### Statistics

Four to six explants in each of three to six separate experiments were evaluated with respect to the expression of various molecular markers on consecutive sections (supplementary material Fig. S9). Explants in which the majority of cells expressed the marker of interest were counted as positive and explants in which the marker was not expressed at all were counted as negative. The statistics are presented as number of explants with positive expression out of the total numbers of explants analyzed (e.g. 8/10).

## Supplementary Material

Supplementary Material
