# Supplementary material for: Neural retina identity is specified by lens-derived BMP signals
Source: Development. 2015 May 15;142(10):1850–9. doi: 10.1242/dev.123653 (PMC4440930; doi:10.1242/dev.123653)
Supplement: Supplementary Material [file supp_142_10_1850__index.html]

Supplementary Material 

# Neural retina identity is specified by lens-derived BMP signals

## DEV123653 Supplementary Material

- Supplementary Material
