# Supplementary material for: Neural retina identity is specified by lens-derived BMP signals
Source: Development. 2015 May 15;142(10):1850–9. doi: 10.1242/dev.123653 (PMC4440930; doi:10.1242/dev.123653)
Supplement: Supplementary Material [file supp_142.10.1850_DEV123653supp.pdf]

Supplementary Figures

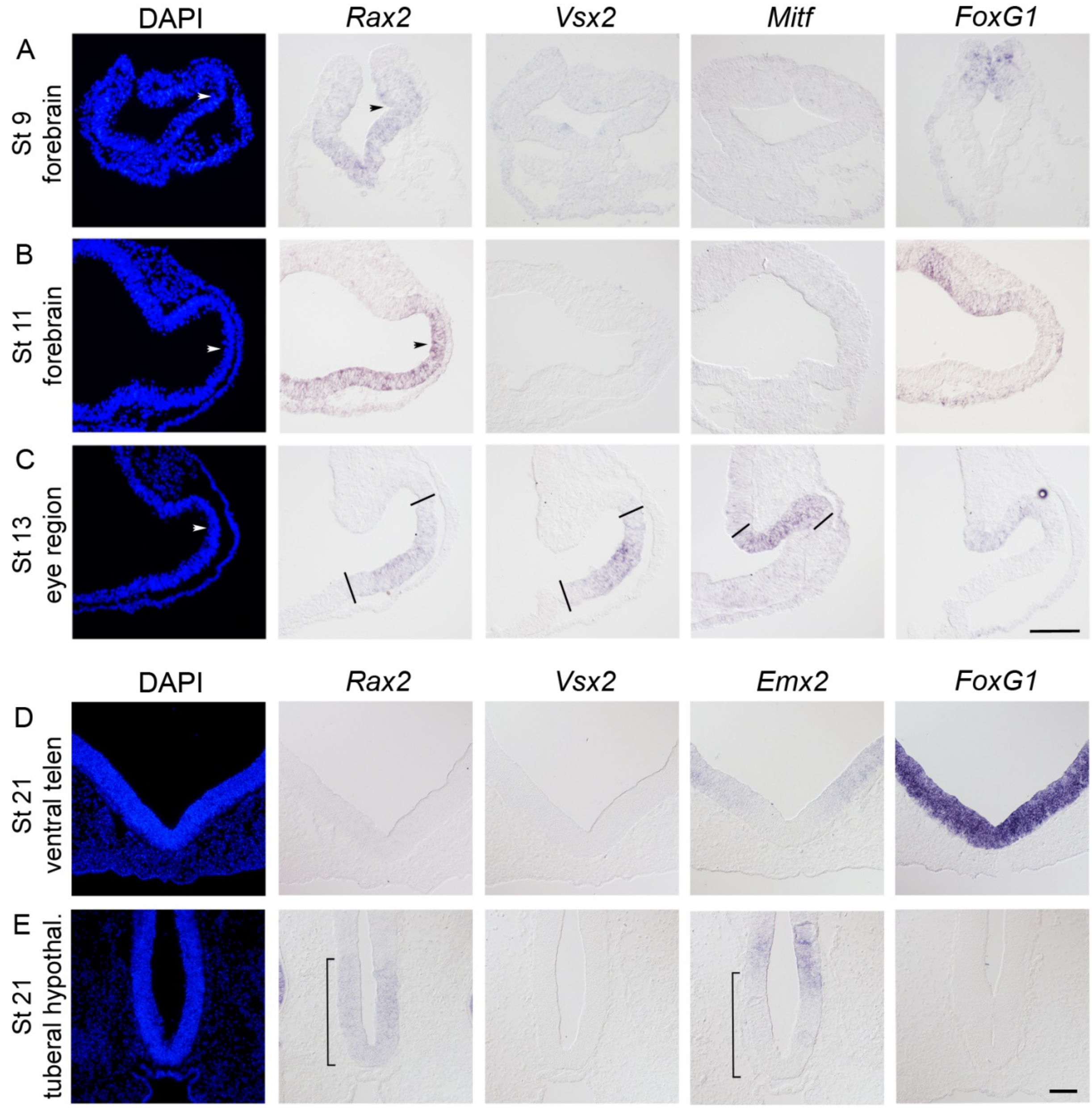

Fig. S1.

Expression patterns of optic vesicle and anterior forebrain markers

Expression patterns of various anterior forebrain markers were analysed at stages 9, 11, 13 and 21 by in situ hybridization on consecutive sections.

(A-C) White arrowheads in first column indicate eye-field cells (A) and optic vesicle cells (B, C).

(A,B) At stage 9 and 11, *Rax2* is expressed within the evaginating eye-field (black arrowheads) and in prospective hypothalamic cells. *FoxG1* expression is confined to prospective telencephalic cells. Neither *Vsx2* nor *Mitf* expression is detected in the forebrain at these stages.

(C) At stage 13, *Vsx2* expression is up-regulated in the neural domain of the optic vesicle together with *Rax2* expression. *Mitf* expression is up-regulated in the prospective RPE of the optic vesicle, where weak *FoxG1* expression also is detected. Neural and RPE domains indicated by black lines.

(D) At stage 21, *FoxG1* is strongly expressed in the ventral telencephalon. *Emx2* expression is not detected in the ventral midline of the telencephalon, and weak *Emx2* expression is observed above the ventral most region of the telencephalon. Neither *Rax2* nor *Vsx2* expression are detected in the ventral telencephalon.

(E) At stage 21, *Rax2* expression is weakly detected in the tuberal hypothalamus (indicated by brackets). Neither *FoxG1*, *Emx2* nor *Vsx2* expressions was detected in this region. Scale bars; 100µm.

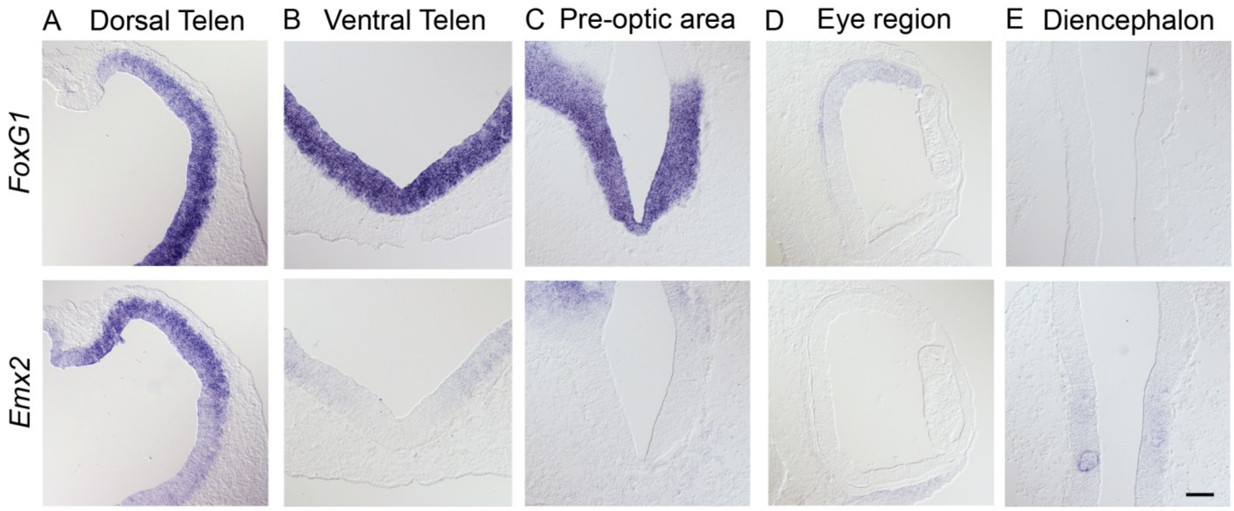

Fig. S2.

Strong *FoxG1* and *Emx2* expression are detected in the dorsal telencephalon

Expression patterns of *FoxG1* and *Emx2* in the forebrain were analysed at stage 21 by in situ hybridization on consecutive sections.

(A) Strong expression of both *FoxG1* and *Emx2* are detected in the dorsal telencephalon.

(B) Strong expression of *FoxG1* is observed in the ventral telencephalon. *Emx2* expression is not detected in the ventral midline of the telencephalon, and weak *Emx2* expression is observed above the ventral most region of the telencephalon.

(C) Weak expression of *FoxG1*, but no *Emx2* expression, is detected in the dorsal periphery of the optic cup.

(D) Strong expression of *FoxG1*, but no *Emx2* expression, is detected in the pre-optic area.

(E) In the walls of the diencephalon, weak expression of *Emx2*, but no *FoxG1* expression, is observed. Scale bar; 100µm.

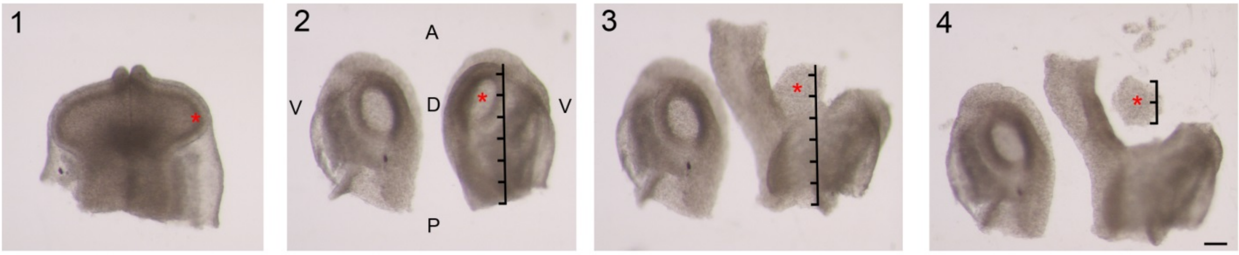

Fig. S3

Dissection of OV/OVL explants

(1) Whole head of a stage 10 embryo.

(2) Head divided into two halves and positioned with the optic vesicles up.

(3) Dissection around the optic vesicle with a tungsten needle.

(4) The optic vesicle/lens (OVL) explant is separated from the rest of the forebrain. Note the small pieces in the upper right corner from fine tuning the OVL dissection. OV explants require removal of the prospective lens ectoderm from OVL explants.

Red asterisk marks the optic vesicle region. A – anterior; P – posterior; V – ventral; D – dorsal. Scale bar; 100μm.

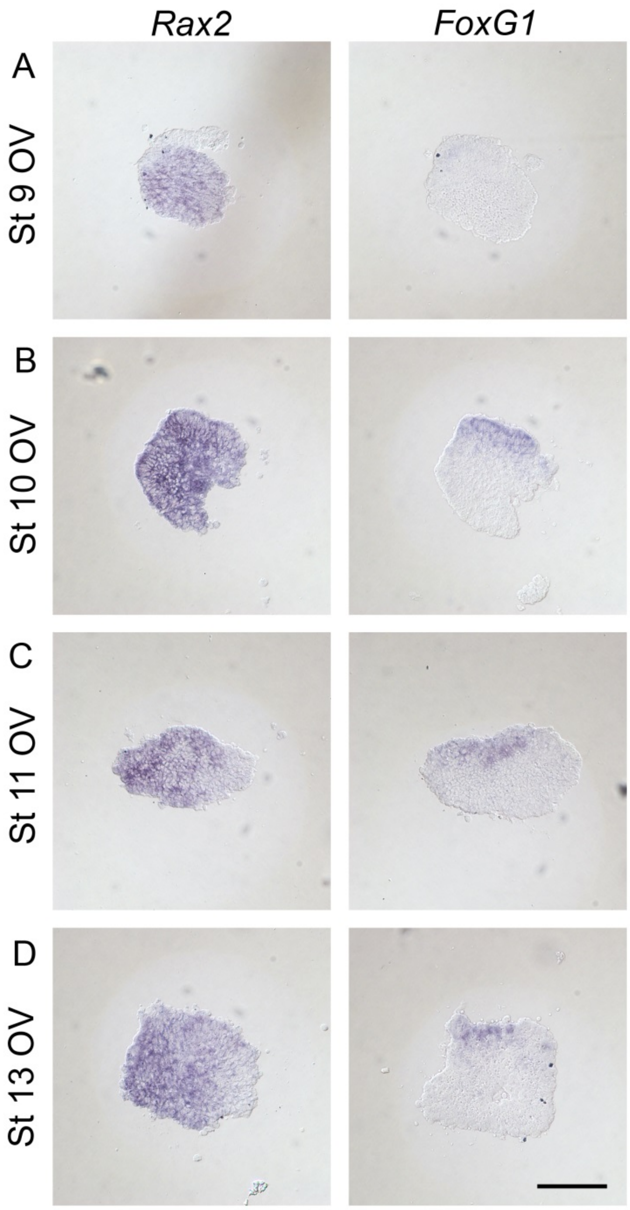

Fig. S4.

Expression of *Rax2* and *FoxG1* in OV explants at the onset of culture

Stage 9, 10, 11 and 13 OV explants fixed at 0 hours (=onset of culture) and analyzed by in situ hybridization on consecutive sections.

(A-D) At 0hr of culture, the majority of stage 9 (n=10), 10 (n=10), 11 (n=5) and 13 (n=5) OV explants consisted of *Rax2*$^+$ cells, and a few *FoxG1*$^+$ cells at one edge of the explants.

Scale bar; 100µm.

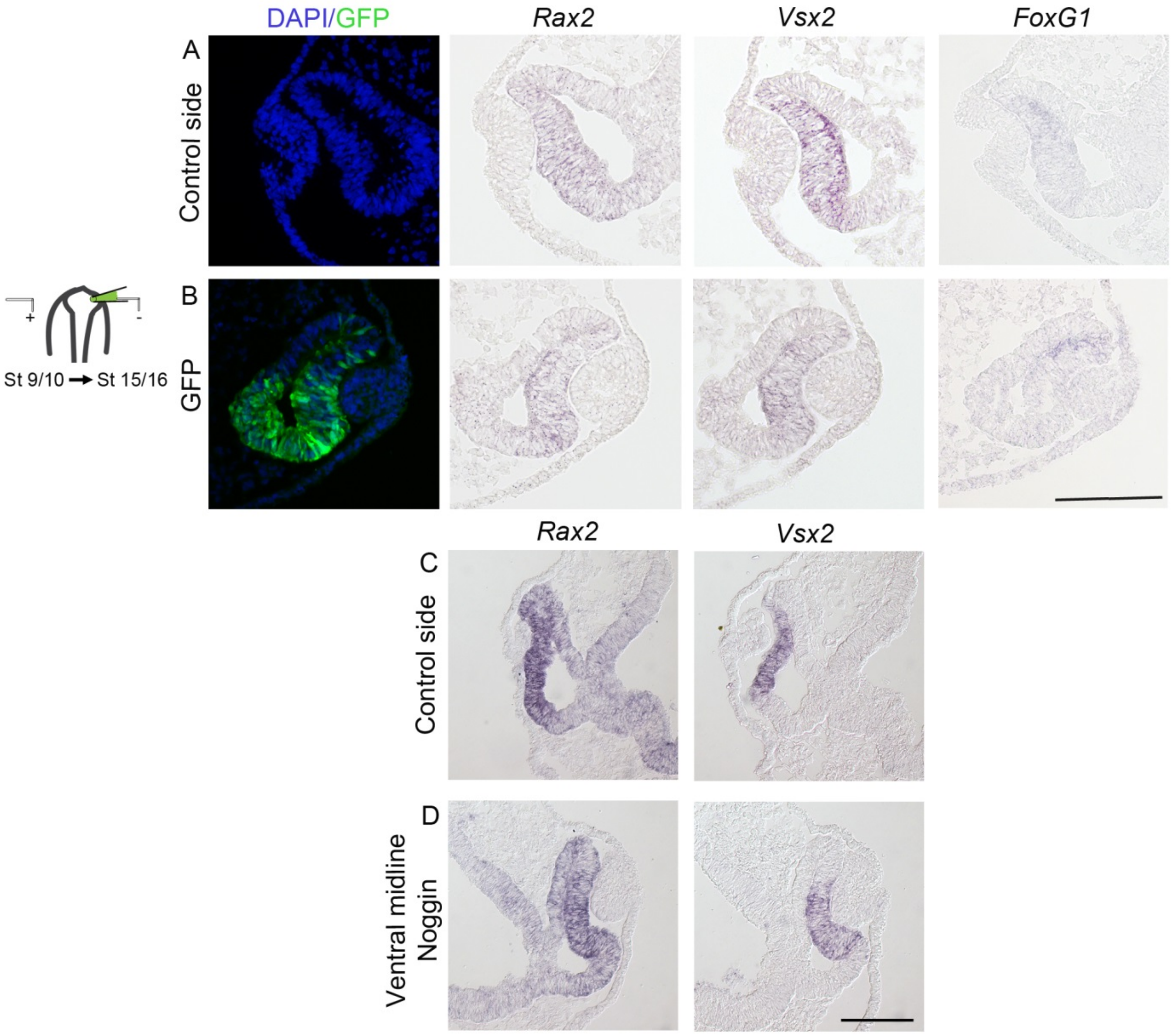

Fig. S5.

In ovo GFP electroporation does not affect retinal development

(A-D) In ovo electroporation of stage 9/10 embryos and cultured to stage 15/16, and thereafter analyzed by immunohistochemistry and in situ hybridization on consecutive sections. Electroporation of the prospective retina (B) or the ventral midline (D) and the control non-electroporated sides (A,C).

(A) Control non-electroporated retina with expression of *Rax2* and *Vsx2* in the neural retina, and weak expression of *FoxG1* in the dorsal part of the neural retina.

(B) Prospective retinal cells electroporated with a GFP vector (8/8) did not disturb retinal morphology, and did not result in altered expression of *Rax2, Vsx2 or FoxG1*.

(C, D) No change in retina morphology or expression of *Rax2* and *Vsx2* was observed after Noggin was electroporated in the ventral midline of the forebrain (n=4). Scale bars; 100µm.

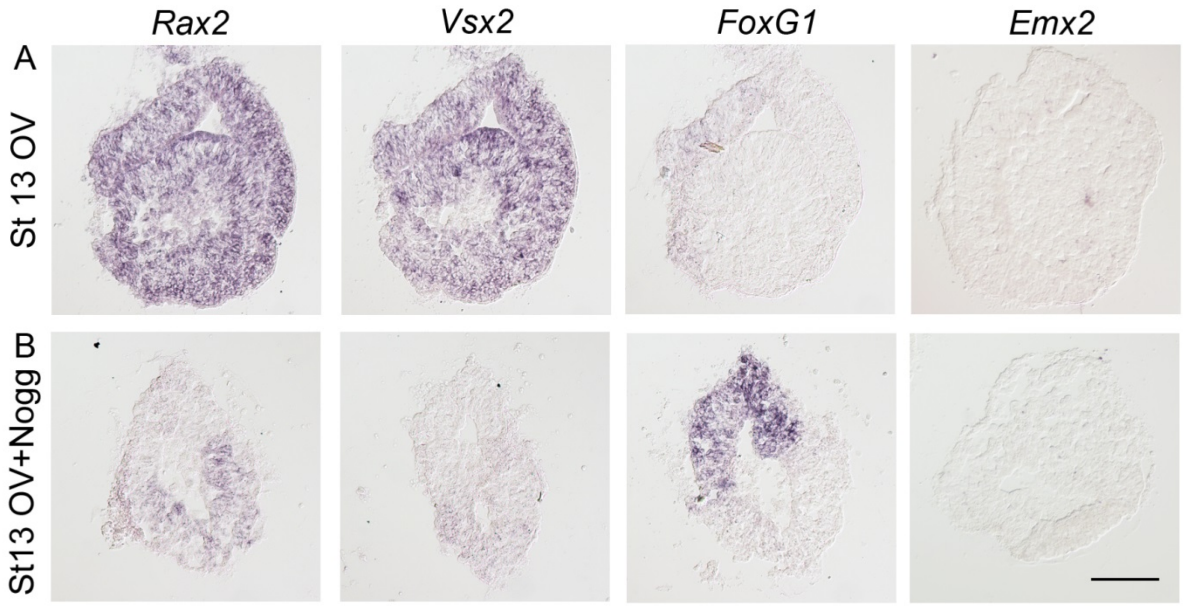

Fig. S6

BMP requirement for the specification of neural retina cells independent of the lens

(A, B) Stage 13 optic vesicle (OV) explants cultured to approximately stage 21 and analyzed by in situ hybridization.

(A) Same as Fig. 1D. Stage 13 OV explants generated *Rax2*$^+$ (15/15) and *Vsx2*$^+$ (15/15) neural retinal cells, and a few *FoxG1*$^+$ cells in a restricted region (15/15), but no *Mitf*$^+$ (0/15) RPE cells or *Emx2*$^+$ (0/15) cells were detected.

(B) Stage 13 OV explants cultured together with Noggin generated two domains; cells in one region strongly expressed *FoxG1* (15/15), but no *Rax2* (0/15), *Vsx2* (0/15) or *Emx2* (0/15), whereas cells in the other domain expressed reduced levels of *Rax2* (15/15), no or weak levels of *Vsx2* (15/15), but no *FoxG1* (0/15) or *Emx2* (0/15). Scale bar; 100μm.

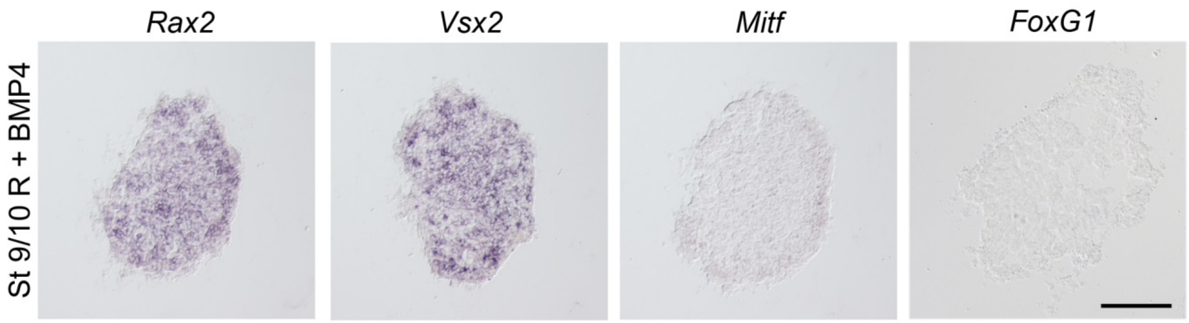

Fig. S7.

High levels of BMP4 do not induce RPE cells

Stage 9/10 R explants cultured to approximately stage 21 and analyzed by in situ hybridization.

In stage 9/10 R explants, BMP4 (35ng/mL) suppressed the generation of *FoxG1*$^{+}$ (0/10) telencephalic cells, and induced *Rax2*$^{+}$ (10/10) and *Vsx2*$^{+}$ (10/10) neural retinal cells, but no *Mitf*$^{+}$ (0/10) RPE cells. Scale bar; 100µm.

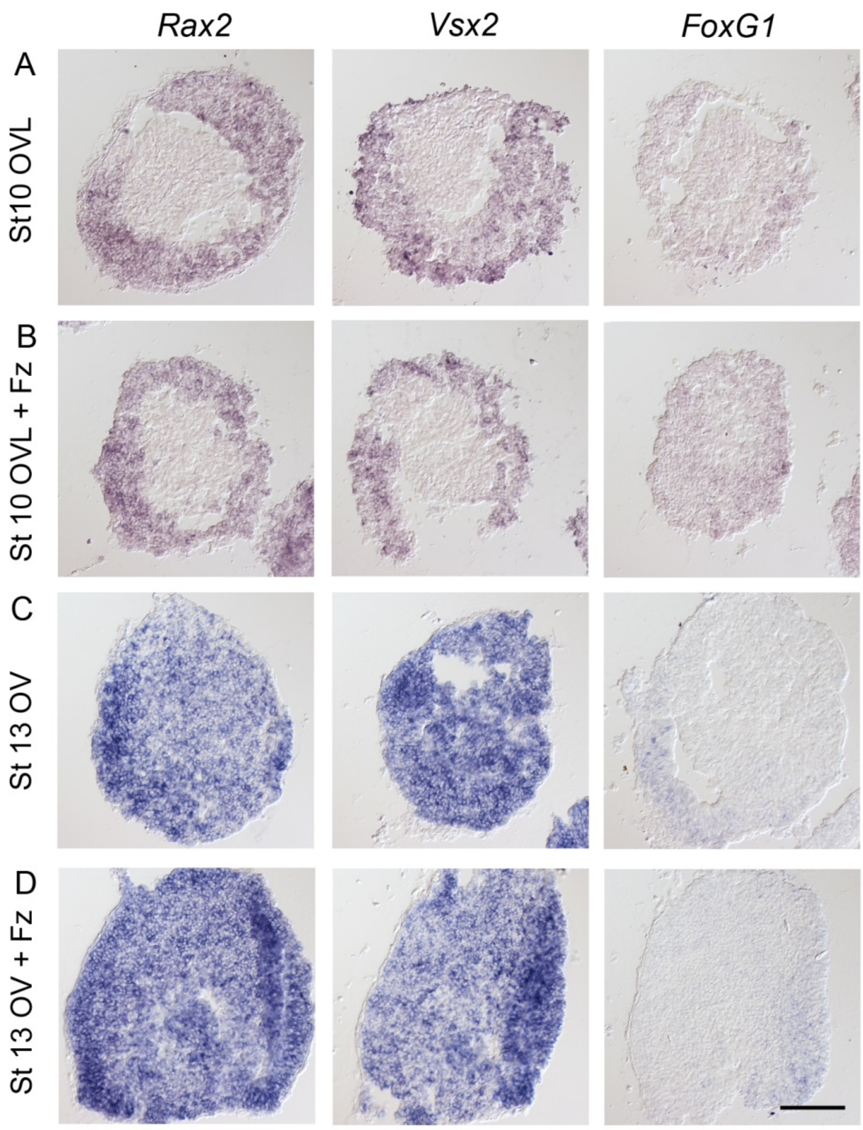

Fig. S8.

Wnt activity is not required for the specification of neural retina cells

(A-D) Stage 10 optic vesicle/prospective lens (OVL) explants, and stage 13 OV explants cultured to approximately stage 21 and analyzed by in situ hybridization on consecutive sections.

(A,B) Both stage 10 OVL explants cultured alone or in the presence of Frizzled generated *Rax2*$^+$ (25/25 OVL; 10/10 OVL+Fz) and *Vsx2*$^+$ (25/25 OVL; 10/10 OVL+Fz) neural retinal cells. No or a few weak *FoxG1*$^+$ (25/25 OVL; 10/10 OVL+Fz) cells were detected.

(C,D) Stage 13 OV explants cultured alone or in the presence of Frizzled generated *Rax2*$^{+}$ (15/15 OV; 10/10 OV+Fz) and *Vsx2*$^{+}$ (15/15 OV; 10/10 OV+Fz) neural retinal cells, and a few *FoxG1*$^{+}$ cells in a restricted region (15/15 OV; 10/10 OV+Fz). Scale bar; 100µm.

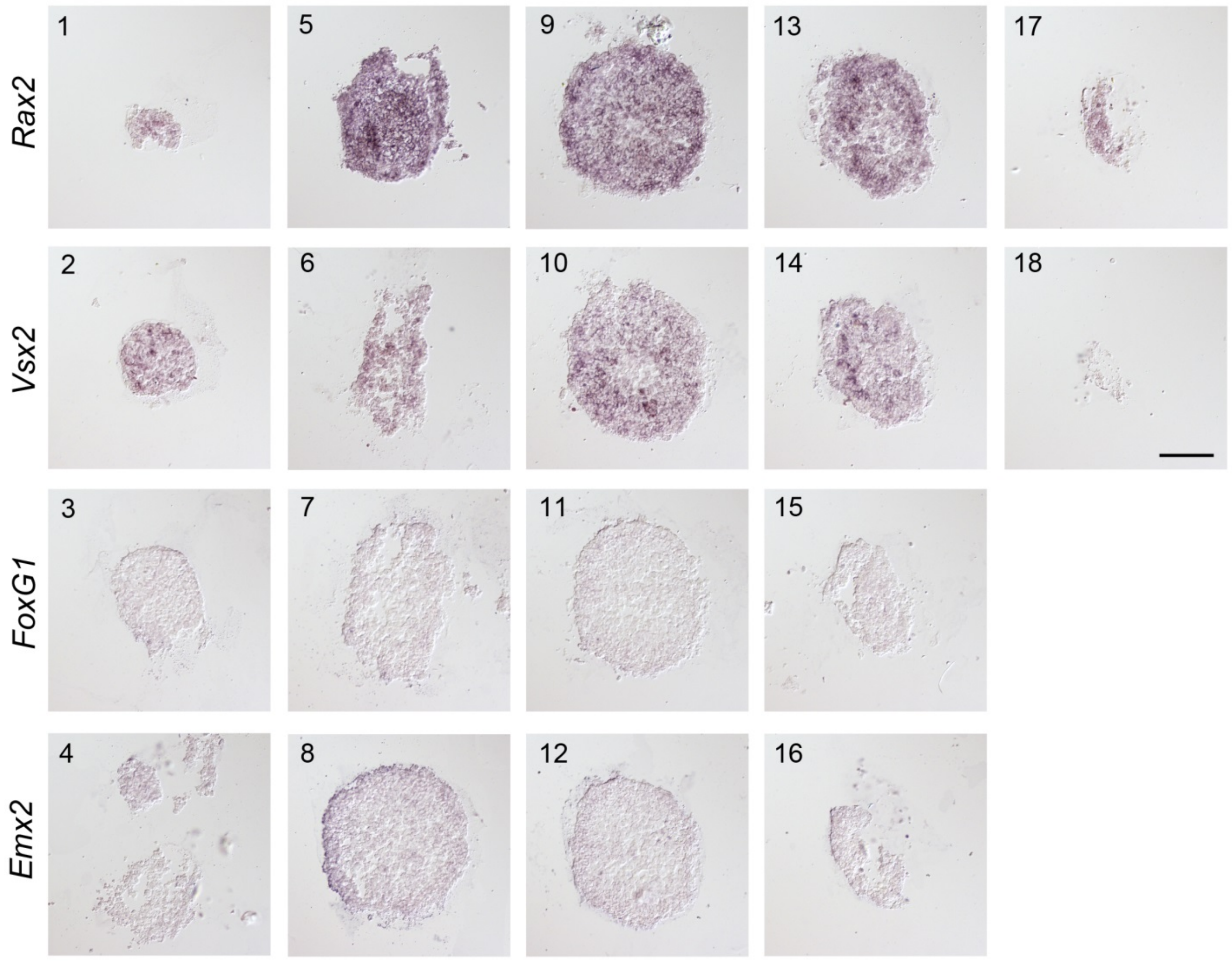

Fig. S9.

Consecutive sections of a st10 OV explant cultured together with BMP4 for 50 hours

An example showing all consecutive sections, labelled 1-18, of a st10 OV explant cultured together with BMP4 for 50 hr and analyzed by in situ hybridization for *Rax2, Vsx2, FoxG1* and *Emx2*.

Supplementary Materials and Methods

Explants

The explants were cultured in vitro in collagen in serum-free OPTI-MEM (GIBCO) containing N2 supplement (Invitrogen) and fibronectin (Sigma) to desired time points. To isolate optic vesicle (OV) and dorsal telencephalic (dT) explants, embryos were first incubated in Hanks balanced salt solution (without Ca^{2+}, Mg^{2+}) (GIBCO) for 3-5 minutes. Then the embryos were transferred to Hanks balanced salt solution (without Ca^{2+}, Mg^{2+}) containing collagenase (Sigma, 1000u/ml) for 2-4 minutes, followed by washing in Hanks balanced salt solution (with Ca^{2+}, Mg^{2+}) containing FCS after which they were transferred to L-15 medium (GIBCO). Noggin and control conditioned medium (CM) were obtained from stably transfected or un-transfected Chinese hamster ovary (CHO) cells (Lamb et al., 1993) and cultured in CHO-S-SFM II media (GIBCO). Soluble Wnt3A and control CM were obtained from stably transfected mouse L cells, and soluble Frizzled 8 and control CM were obtained from HEK-293 cells transfected with *mFrz8CRD* or *LacZ* reporter construct (Hsieh et al., 1999). The activity of Frizzled CM was tested on proven assays (Patthey et al., 2009). Noggin CM was used at an estimated concentration of 50 ng/ml, and Wnt3A at an estimated concentration of 30 ng/ml. BMP4 (R&D Systems) was used at 3.5-35ng/ml and FGF8 (R&D Systems) was used at 250ng/ml together with 0.5µg/ml heparin (Sigma). SU5402 (Calbiochem) was used at 5µM. Explants cultured in the presence of control CM generated the same combination of cells as explants cultured alone (data not shown).

In ovo electroporation

Vectors used for electroporation were: pCAβ-*EGFP*-m5 (1 µg/µl) and pMiwIII –Noggin (1 µg/µl) (Timmer et al., 2002). The DNA-constructs were transferred using an Electro Square

Porator ECM 830 (BTX.Inc) by applying 3 pulses (9-18 Volts, 25ms duration,) at 1-s intervals. After electroporation the embryos were cultured in ovo to stage 15-16. Electroporated domains in the Noggin-electroporated embryos were compared to the corresponding region of the non-electroporated side as well as control GFP-electroporated embryos.

In situ hybridization and Immunohistochemistry

For the use of in situ RNA hybridization and immunohistochemistry, embryos were fixed in 4% paraformaldehyde (PFA) in phosphate buffered saline (PBS) for 1.5 hours and explants for 25-30 minutes at 4°C. In situ hybridization was performed using the following Dig-labelled chick probes *Emx2*, *FoxG1* (McCarthy et al., 2001), *Fgf8*, *Mitf* (Mochii et al., 1998), *Rax2* (Sanchez-Arrones et al., 2009), *Vsx2* (Chen and Cepko, 2000). Antibodies used were: anti-sheep δ-crystallin (Beebe and Piatigorsky, 1981) and anti-mouse HuC/D (Molecular Probes). Nuclei were stained using DAPI (Sigma).

Supplementary References

Beebe, D. C. and Piatigorsky, J. (1981). Translational regulation of delta-crystallin synthesis during lens development in the chicken embryo. *Developmental biology* **84**, 96-101.

Chen, C. M. and Cepko, C. L. (2000). Expression of Chx10 and Chx10-1 in the developing chicken retina. *Mechanisms of development* **90**, 293-297.

Hsieh, J. C., Rattner, A., Smallwood, P. M. and Nathans, J. (1999). Biochemical characterization of Wnt-frizzled interactions using a soluble, biologically active vertebrate Wnt protein. *Proceedings of the National Academy of Sciences of the United States of America* **96**, 3546-3551.

Lamb, T. M., Knecht, A. K., Smith, W. C., Stachel, S. E., Economides, A. N., Stahl, N., Yancopolous, G. D. and Harland, R. M. (1993). Neural induction by the secreted polypeptide noggin. *Science (New York, N.Y* **262**, 713-718.

McCarthy, M., Na, E., Neyt, C., Langston, A. and Fishell, G. (2001). Calcium-dependent adhesion is necessary for the maintenance of prosomeres. *Developmental biology* **233**, 80-94.

Mochii, M., Mazaki, Y., Mizuno, N., Hayashi, H. and Eguchi, G. (1998). Role of Mitf in differentiation and transdifferentiation of chicken pigmented epithelial cell. *Developmental biology* **193**, 47-62.

Patthey, C., Edlund, T. and Gunhaga, L. (2009). Wnt-regulated temporal control of BMP exposure directs the choice between neural plate border and epidermal fate. *Development (Cambridge, England)* **136**, 73-83.

Sanchez-Arrones, L., Ferran, J. L., Rodriguez-Gallardo, L. and Puelles, L. (2009). Incipient forebrain boundaries traced by differential gene expression and fate mapping in the chick neural plate. *Developmental biology* **335**, 43-65.

Timmer, J. R., Wang, C. and Niswander, L. (2002). BMP signaling patterns the dorsal and intermediate neural tube via regulation of homeobox and helix-loop-helix transcription factors. *Development (Cambridge, England)* **129**, 2459-2472.
